# Determination of Band Structure of Naturally Occurring Goethite with Al Substitution: A Case Study of Zhushan Iron Zone

**DOI:** 10.3390/ma15041465

**Published:** 2022-02-16

**Authors:** Yan Shao, Guofeng Hu, Zihao Liu, Xiaoming Xu, Mengqi Zhang, Cong Ding, Yan Li

**Affiliations:** 1 Wuhan City Environment Protection Engineering Limited Company, Wuhan 430205, China; 37004@ccepc.com (Y.S.); 54001@ccepc.com (G.H.); 10142@ccepc.com (Z.L.); 2 School of Earth and Space Sciences, Peking University, Beijing 100871, China; mengqizhang@pku.edu.cn (M.Z.); cding_pku@163.com (C.D.); lyanpku@163.com (Y.L.)

**Keywords:** goethite, Al substitution, band structure, valence band, photocatalysis

## Abstract

The photocatalytic property of Fe oxide minerals has long been considered to play an important role in shaping modern terrestrial environments. However, due to the complexity of natural settings, a precise determination of the band structure of natural goethite has not been achieved. In this work, the mineralogical characteristics of natural goethite samples obtained from Zhushan, China, were systematically studied through X-ray diffraction, transmission electron microscopy, X-ray energy dispersive spectroscopy, and X-ray fluorescence spectroscopy. Afterward, the band structure for both natural and synthetic goethite samples was determined by synchrotron-based X-ray absorption and emission spectra and photoelectron spectroscopy. The band gap of natural goethite (2.25 eV) was narrower than that of its synthetic counterpart (2.55 eV), and the valence band position of natural goethite was slightly lifted (−5.06 eV) compared to that of synthetic goethite (−5.38 eV). Al doping in natural goethite crystal, as revealed by the mineralogical tests, was the main reason that contributed to this difference. The theoretical calculation showed the narrowed band gap was caused by the contribution of Al-2p orbits at the top of the valence band. Therefore, free electrons can be created under light irradiation with a shorter wavelength. The experiments showed that natural goethite can photo-catalytically degrade methyl orange, and the degradation efficiency was better (47.5%) than that of the synthetic goethite group (31.5%). This study, for the first time, revealed the band structure and confirmed the photocatalytic properties of natural goethite, which should play an important role in surface substance evolution and elemental cycling.

## 1. Introduction

Fe is the most abundant transition metal element on the Earth’s surface and can form a diversity of oxide minerals in different weathering environments. So far, more than 16 Fe (hydro)oxide minerals have been identified in different geological settings [1], such as rock coatings [2], soil cutan [3], iron ore deposits [4], water surface films [5], and freshwater ferromanganese nodules [6]. For a long time, natural Fe oxide minerals have played important roles in social-economic development due to being mineral resources [7]. Fe oxide minerals have been found to possess the capability of recording climate change [8,9] and of serving as good heavy metal or tonic organics scavengers [10,11] because of their natural occurrences.

Fe oxide minerals are also semiconducting materials with good responses to solar light irradiation. Commonly, the band gap of iron oxide minerals is 2.0–2.5 eV [12]. This means these widespread natural Fe oxide minerals can form active photo-generated hole–electron pairs to promote redox reactions on the Earth’s surface under irradiation with a wavelength shorter than 615 nm, thus playing important roles in shaping terrestrial environments. For example, Georgiou et al. [13] reported reactive oxygen species formed on goethite surfaces could promote carbon cycling in desert regions. Lan et al. [14], Xu et al. [15], and Jung et al. [16] found Fe oxide minerals can promote Mn^2+^ oxidation, implicating a new pathway for Mn(III/IV) minerals formation. Many types of organic matter, including bisphenol, methylene blue, rhodamine, and acetaldehyde, can be photo-catalytically degraded in the presence of Fe oxide minerals [17,18,19]. All these lines of evidence show the potential impact of Fe oxide minerals involved in photochemical reactions on the surface geochemical process.

Among different chemical forms of Fe oxide minerals, goethite FeOOH is one of the most representative on the Earth’s surface. Natural minerals are rarely pure and pristine. Their inherent chemical complexity can cause changes in their band structure, which are reflected during photocatalytic activity. Furthermore, particle size may be another factor that influences the band structure of goethite [19,20]. Synthetic goethite is commonly smaller than natural goethite [21]. So, goethite in natural forms may have a diverse electronic structure compared to its laboratory counterpart. However, a detailed understanding and interpretation of the electronic structure and photocatalytic performance of natural goethite is not available, which has greatly restricted its application in the field of semiconducting materials.

In this study, natural and synthetic goethite samples were prepared for comparative research. The crystal structure, micro-morphology, and chemical composition of natural samples were examined by X-ray diffraction (XRD), transmission electron microscopy (TEM), X-ray energy dispersive spectroscopy (EDS), and X-ray fluorescence spectroscopy (XRF). The experimental determination of band structure parameters was achieved by using synchrotron-based O K-edge X-ray absorption and emission spectroscopy (XAS and XES) and photoelectron spectroscopy (PS). Based on the mineralogical characterization, density functional theory (DFT) was employed to theoretically outline the electronic structure of natural and synthetic goethite. Photochemical degradation of methyl orange (MO) by different goethite samples was conducted to compare their actual photocatalytic performance. The mineralogical tests, photochemical experiments, together with theoretical simulation can provide some insights on the band structure of natural goethite and deepen the understanding of goethite-involved photochemical reactions in terrestrial environments.

## 2. Materials and Methods

### 2.1. Sample Preparation

The natural goethite sample used in this study was collected from the surface Fe coatings in the Zhushan Iron Zone, Hubei Province, China. After drying the sample at a low temperature of 40 °C, it was ground using a high-purity quartz rod in an agate mortar and then sieved to achieve a particle size of less than 25 μm. A synthetic goethite sample with a size of 25 nm was purchased from Alfa Aesar Reagents Ltd. (Ward Hill, MA, USA). All other chemicals (e.g., deionized water, ethyl alcohol, and sodium sulfate) were purchased from Sinopharm Chemical Reagent Beijing Co., Ltd. (Beijing, China).

### 2.2. X-ray Diffraction (XRD) Analysis

The phase identification of natural goethite samples was carried out by an X-ray powder diffractometer (D/max-rA, Rigaku Industrial Corporation, Osaka, Japan) using Cu Kα1 radiation (λ = 1.5406 Å). The scanning speed of angle 2θ was 0.5°/min with a scanning range of 5°–85°. During the test, the applied voltage was 40 kV and the applied current was 100 mA. The diffraction data were analyzed and processed with Jade 6.0 software.

### 2.3. X-ray Fluorescence (XRF) Spectroscopy Analysis

The natural goethite sample powder was placed in a porcelain crucible, which was dried to a constant weight and then baked in an oven at 105 °C for 12 h. The sample was cooled in a glass dryer to room temperature and then calcined in a muffle oven at 800 °C for 2 h. After taking it out, it was cooled in a dryer to room temperature to calculate the loss on ignition (LOI) of the sample. The sample powder was then placed in a tablet press to make the tablet required for X-ray fluorescence spectroscopy (XRF-1800, Shimadzu Corporation, Kyoto, Japan) analysis.

### 2.4. Transmission Electron Microscopy (TEM) Analysis

A transmission electron microscope (JEM-2100F, JEOL, Akishima, Japan) equipped with an X-ray energy dispersive spectrometer was used to analyze the crystallographic characteristics of natural goethite at the nanoscale. The goethite powders were dissolved in the alcohol solvent, and 0.1 mL of the mixed alcohol solution was ultrasonically dispersed and then dropped onto copper mesh with a diameter of 2.5 mm. The excess alcohol was absorbed by filter paper on the other side of the grid. The copper mesh was then placed on the sample table of the transmission electron microscope. During the test, the operating voltage was 200 kV and the spatial resolution of a single point was 0.19 nm. The experiment data were analyzed and processed by Digital Micrograph v3.9 software.

### 2.5. Synchrotron X-ray Absorption and Emission Spectroscopy (XAS and XES) Analysis

The band gaps of both natural goethite and synthetic goethite samples were obtained by measuring the oxygen K-edge absorption and emission spectra on a 10ID-2 beamline at the Canadian Light Source. The monochromatic radiant light emitted from the undulator was incident upon the powder sample mounted on the carbon strip. The sample plate was at 60° with the incident beam. The spatial resolution of XAS was 0.25 eV and the spatial resolution of XES was 0.55 eV. The experiment was carried out at vacuum pressure lower than 10^−9^ torr with a slit width of 25 mm. The absorption spectrum data were obtained in the form of total electron yield and calibrated according to the standard spectral data of goethite [22]. The emission spectrum data were calibrated by testing the elastic lines with known incident energy.

### 2.6. Photoelectron Spectroscopy (PS) Analysis

The highest occupied molecular orbital level of the goethite samples was measured by a photoelectron spectrometer (AC-2, Riken Keiki, Saitama, Japan). The light source was a xenon lamp with a power of 800 nW, and the splitter was a grating monochromator. Sample powders were pressed to make a tablet for the analysis. The initial voltages of suppression grid, quenching grid, and anode were 80, 100, and 2900 V, respectively. The spatial resolution of the work function was 0.2 eV and the step size was 0.05 eV. The quenching and counting times were 5 ms and 10 s, respectively.

### 2.7. Density Functional Calculations

The theoretical calculations were performed by density functional theory, using generalized gradient approximation (GGA) to describe the exchange-correlation potential [23], implemented in the VASP-PAW package. The cutoff energy for the expansion of the plane wave was 500 eV. A 4 × 4 × 4 k-mesh was used in the Brillouin zone according to the Monkhorst-Pack scheme. The convergence accuracy of the forces acting on each atom was less than 0.01 eV/Å.

A spin-polarized treatment was adopted for accurate goethite (FeOOH) DFT calculations because Fe exhibits a variety of states in Fe oxides. In this study, calculations for all goethite models were conducted with spin polarization and exhibited antiferromagnetic ordering among irons [24]. The electron configurations were 1s^2^2s^2^2p^6^3s^2^3p^6^3d^6^4s^2^ for Fe and 1s^2^2s^2^2p^4^ for O. The partial density of states (DOS), which describes the qualitative contribution of specific angular momentums of atoms (e.g., Fe-3d, O-2p) to the total number of electronic states corresponding to each energy level, was determined by the projection of plane-wave eigenfunctions onto pseudo-atomic basis sets.

### 2.8. Photochemical Experiments

The photochemical experiments were conducted to compare the catalytic performance of different gothite samples. A three-electrode system was used for the photochemical experiments of methyl orange degradation. A platinum sheet was used as the counter electrode. Goethite powders were pasted on FTO conductive glass to serve as the working electrode. A calomel electrode was used as the reference electrode. The electrolyte was 0.5 M Na_2_SO_4_. A Xe lamp was used as the simulated sunlight source, and the light intensity was 150 mW/cm^2^. During the experiment, the initial concentration of MO was 25 ppm, and the samples were taken out from the reactor every 30 min to test the residual concentration of MO. The concentration of MO was acquired by testing its maximum absorption peak at 495 nm with a spectrophotometer (Evolution 220, Thermo Fisher, Waltham, MA, USA).

## 3. Results

### 3.1. XRD Analysis

**Figure 1:**
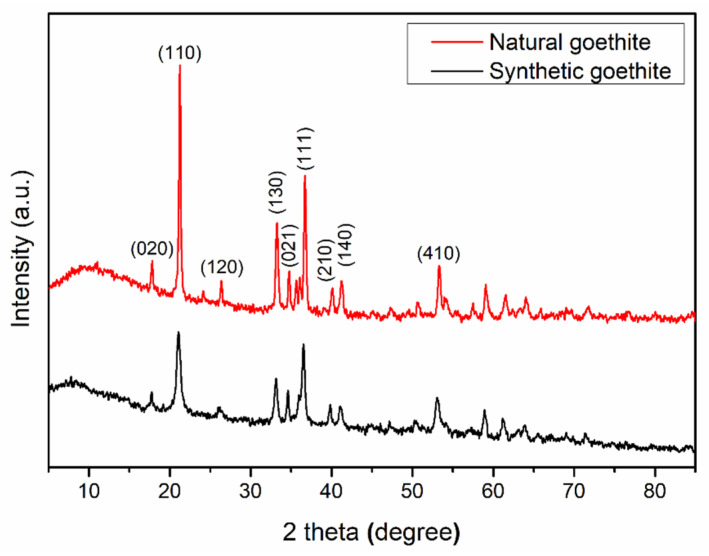
XRD pattern of natural goethite and synthetic goethite powders.

The crystal structures of natural goethite and its synthetic counterpart were analyzed by XRD, and the XRD results of samples are shown in Figure 1. The patterns show that their main composition is goethite, which is consistent with the diffraction peak position and intensity of standard goethite (JCPDS: 00-003-0249). The main diffraction peaks of the natural sample are located at 17.7°, 21.2°, 26.4°, 33.2°, 34.7°, 36.1°, 40.0°, 41.2°, and 53.1°, which can be attributed to the goethite crystal face diffraction of (020), (110), (120), (130), (021), (111), (210), (140), and (410), respectively. However, there are minor peaks at 24.2° and 26.6° in the natural goethite samples (Figure 1), which may correspond to the (012) plane diffraction peak of hematite and (101) plane diffraction peak of quartz, indicating minimal impurities in the natural goethite samples.

### 3.2. TEM Analysis

**Figure 2:**
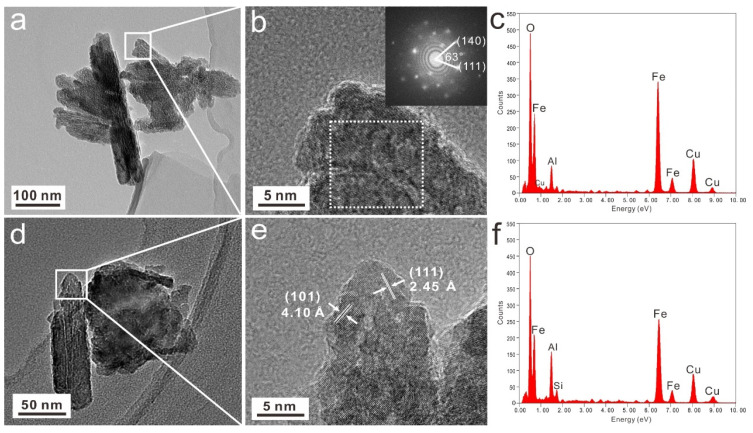
TEM morphology (**a**,**d**), lattice photograph (**b**,**e**), and EDS data (**c**,**f**) of natural goethite powders.

Under TEM, natural goethite crystals were rod-shaped, 100–300 nm in length, and 30–50 nm in width (Figure 2a,d). In the high-resolution image, regions with a well-developed crystal domain were picked, in which two groups of crystal faces with a d spacing of 2.45 Å and 4.10 Å could be discriminated. These can be attributed to the goethite crystal faces of (111) and (110), respectively (Figure 2b,e). The EDS data show that natural goethite crystals contain a small amount of Al impurity, indicating that Al can replace Fe into goethite lattice in the form of isomorphic substitution (Figure 2c,f). This observation is consistent with the results of previous studies in the literature [25,26,27].

### 3.3. XRF Analysis

The results of the chemical composition of natural goethite samples as revealed by XRF are presented in Table 1. The total iron content of the sample is 77.71–84.79%. In addition, it contains a small amount of SiO_2_ (1.32–4.05%) and Al_2_O_3_ (1.06–3.21%) impurities (Table 1), and contents of other elements are very small or below the detection limit. The results of XRF are consistent with XRD and TEM analysis, indicating that there is a small amount of quartz particles in the natural goethite sample, and Al can be incorporated into goethite lattice and co-exist with it.

### 3.4. XAS and XES Analysis

Due to the complex chemical composition of natural semiconducting minerals, the absorption peak caused by the impurity level may overwhelm the intrinsic absorption of the sample. Therefore, it is difficult to determine the band gap of natural semiconducting materials by traditional test methods such as UV-DRS. Sherman first proposed analyzing the band structure of Fe/Mn semiconductors by using the oxygen K-edge absorption spectrum and emission spectrum [12]. For transition metal oxides, the valence band (VB) consists of O-2p orbits and 3d orbits of metal atoms in the occupied state. The conduction band (CB) consists of unoccupied 3d orbits of metal atoms. The oxygen K-edge absorption spectrum indicates an electron transfer from O-1s to O-2p orbits. In the metal oxides, the O-2p orbits are not fully occupied, and these empty O-2p orbits can hybridize with the 3d, 4s, and 4p orbits of metal atoms to form covalent bonds. The oxygen K-edge emission spectrum is generated by the excited electrons falling back from O-2p to O-1s orbits, so that the emission spectrum can indicate the energy of the VB [12]. The band gap of the sample can be determined by measuring the energy difference between the absorption spectrum and the emission spectrum. This method is not limited by the complex chemical composition of the sample, so it is suitable for natural semiconducting minerals.

The X-ray emission and absorption spectra reflect the local density of occupied and unoccupied electronic states, respectively. Therefore, the band gap, the difference in energy between the highest occupied and lowest unoccupied electronic states, can be obtained by merging the XAS and XES spectra.

**Figure 3:**
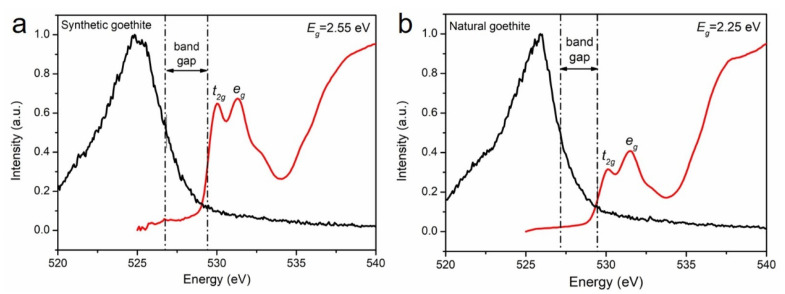
Oxygen K-edge X-ray absorption spectra (red line) and emission spectra (black line) of synthetic goethite (**a**) and natural goethite (**b**).

Figure 3 shows the X-ray absorption and emission spectra of synthetic and natural goethite. The absorption spectrum indicates the electron transfer from O-1s to Fe-3d orbits, and the energy of the absorption spectrum splits, labeled as t2g and eg energy state, which is caused by the splitting of the crystal field of Fe-3d orbits. The emission spectrum indicates the electron transfer from O-2p to O-1s orbits. In order to maintain consistency, energy at the inflection point of the absorption spectrum and emission spectrum is selected as the energy of the CB and VB [12]. The results show the band gap of synthetic goethite is 2.55 eV, which is consistent with the value in the literature [28], while the band-gap of natural goethite is 2.25 eV.

### 3.5. PS Analysis

**Figure 4:**
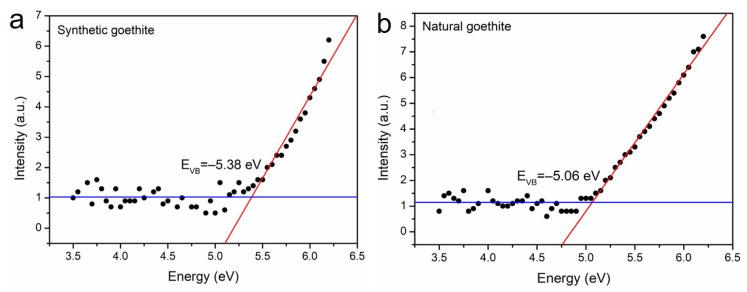
Photoemission spectra of synthetic goethite (**a**) and natural goethite (**b**).

The absolute energy position of VB and synthetic and natural goethite samples in the absolute vacuum scale was measured by a photoelectron spectrometer. For synthetic goethite, the top position of its VB is −5.38 eV (Figure 4a). The top VB position of the natural goethite sample is −5.06 eV (Figure 4b), which is slightly higher than that of synthetic goethite. Based on the band-gap values revealed by XAS and XES analysis (Figure 3), it can be inferred that the bottom position of CB of synthetic goethite is −2.83 eV, and that of natural goethite is −2.81 eV.

### 3.6. Density Functional Calculations

#### 3.6.1. Building the Crystal Models

The model of the goethite unit cell was built by using experimental values [29]. The unit cell has a space group of Pnma with lattice parameters of a = 9.91 Å, b = 3.01 Å, and c = 4.58 Å. Optimized lattice parameters are in good agreement with the experimental values, which are listed in Table 2.

**Figure 5:**
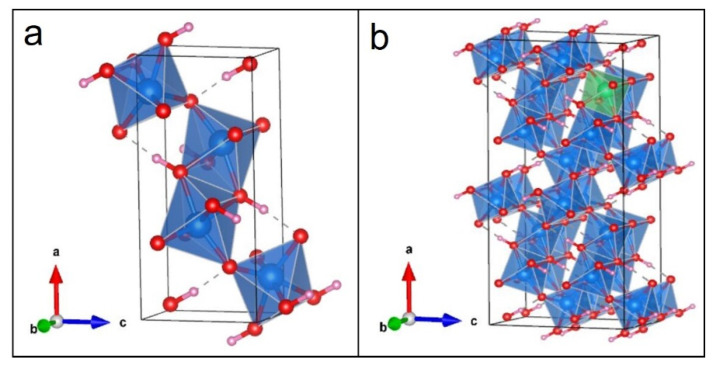
Structure of the goethite unit cell (**a**) and Al-doped goethite supercell (**b**). (Blue, red, pink and green spheres denote Fe, O, H and Al atoms, respectively.)

As revealed by the mineralogical characterization, Al impurity is the most distinctive chemical feature of natural goethite, which was chosen here to explore its influence on the band structure. To obtain the electronic structures of Al-doped goethite, a 2 × 2 × 2 goethite supercell was built using the above optimized lattice parameters, and an Fe atom was substituted by Al with a doping amount of 6.25% (Figure 5). Commonly, the band-gap value calculated using GGA is generally smaller than the experimental value. More precise band-gap estimates can be achieved with DFT + U method [30]. In this study, for Fe, the U value was set as 6 eV and the J value was set as 0.9 eV [31]. Optimized geometries of Al-goethite with labeled atoms can be seen in Figure S1.

#### 3.6.2. Electronic Structure of Different Models

The partial DOS of the goethite unit cell shows spin-dependent hybridization mainly between Fe-3d and O-2p states. More specifically, the contribution of the O-2p state is dominant in the top VB while that of Fe-3d state is dominant in the CB. The spin-up and -down parts of the goethite unit cell are completely symmetrical, which is consistent with the findings of a previous study where pure goethite FeOOH exhibited antiferromagnetic properties [24]. The hybridization between Fe-3d and O-2p orbits indicates that the band-gap should correspond to the charge transfer in O-2p → Fe-3d states. Because of the strong Fe-O covalency, electron transfer across the forbidden band also involves Fe-3d → Fe-3d intraconfigurational-type transitions.

**Figure 6:**
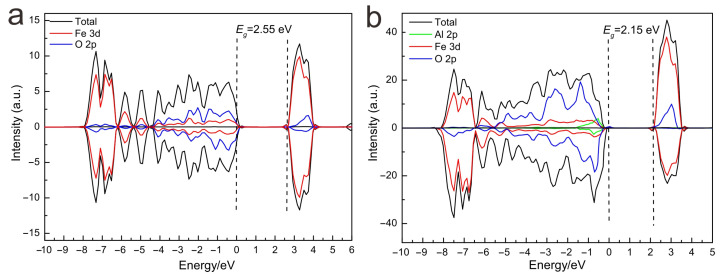
Total and projected DOS of pure goethite cell (**a**) and Al-doped goethite supercell (**b**).

As shown in Figure 6, pure goethite FeOOH possesses a band gap of 2.55 eV, while the band gap of Al-doped goethite is reduced to 2.15 eV. Compared to the DOS of pure goethite, the spin-up and -down parts of Al-goethite are no longer symmetrical, which means the introduction of Al may change the magnetic property of goethite. The atomic Bader charge indicates that Al and Fe should have a +3 oxidation state. The O atoms around Al ions have an additional negative charge with respect to pure goethite. The charge density suggests that the zone surrounding the Al ion undergoes depletion of electronic charge and some electron redistribution in the neighboring O atoms. This behavior can produce small magnetization in these O ions. The contribution of O-2p and Fe-3d states to the VB and CB is similar in the two cases. Specifically, Al doping introduces hybridization of Al-2p and O-2p orbits in the topmost part of the VB, which slightly lifts the VB position to higher energy. In contrast, the CB position remains unchanged (Figure 6b). Therefore, the band-gap shrink of Al-doped goethite is ascribed to the hybridization of Al-2p and O-2p orbits in the VB, which is consistent with the PS analysis (Figure 4).

### 3.7. Photochemical Degradation of Methyl Orange

**Figure 7:**
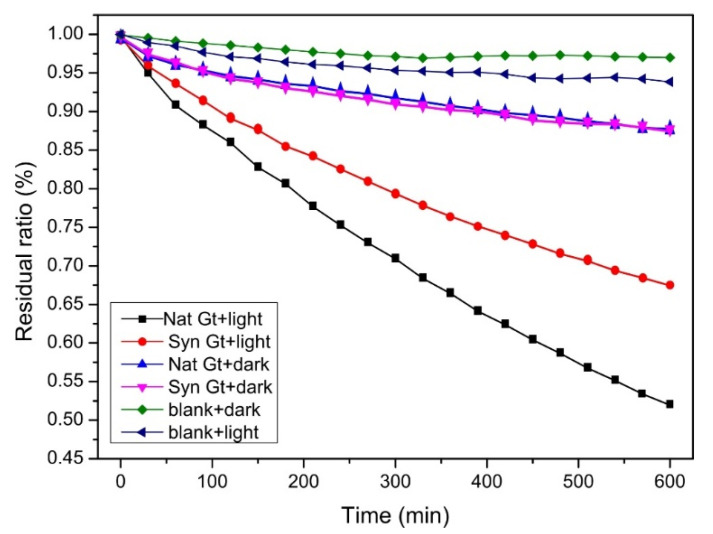
Curves of MO degradation by synthetic goethite and natural goethite electrode.

The content of MO decreases with time in different groups. Notably, MO can be adsorbed by the quartz reactor in the blank group or adsorbed by mineral samples in the dark group. This kind of physical process can give rise to a removal ratio of ~10% at most (Figure 7). However, when simulated solar light irradiation is introduced to this system, the content of MO drops sharply as a function of time, which means photo-oxidation is the main process that contributes to the degradation of MO. Specifically, free electrons can be excited from the VB of the semiconducting goethite to the CB, where holes with oxidation capacity are generated, and MO molecules can be captured and consumed by these holes or reactive oxygen species [13]. In the experiments, synthetic and natural goethite showed similar degradation trends and achieved a degradation ratio of 31.5% and 47.5%, respectively (Figure 7), implicating that natural goethite should possess better photocatalytic properties than its synthetic counterpart.

## 4. Discussion

As a Fe mineral phase that is widespread in many terrestrial weathering environments, goethite FeOOH is reported to carry out different photochemistry-related reactions, playing important roles in surface elemental cycling and substance evolution. However, natural minerals are not pure in chemistry and possess complicated components such as doped atoms and lattice vacancies. Therefore, a comprehensive understanding of the band structure of naturally occurring goethite is necessary.

In this study, the experiments were performed to reveal the ban -gap and electronic structure of natural goethite and its pure synthetic counterpart. The XAS and XES analyses revealed that the band gap of natural goethite is 2.25 eV, which is less than the band gap of synthetic goethite (2.55 eV), which means that natural goethite can absorb a spectrum with a wavelength less than 553 nm. Photocatalysis can be more easily triggered under visible light irradiation. Commonly, the band structure difference can be caused by many factors. The particle size of natural goethite used in this study was about 50–300 nm (Figure 3), larger than its synthetic counterpart (~25 nm). Previous studies demonstrated that the band-gap value of goethite FeOOH decreases with increasing particle size from 10 to 40 nm [20]. Therefore, natural goethite, which is formed over long periods in geological settings, should inherently possess a narrower band gap.

Furthermore, isomorphous substitution, which is common in natural samples, is another factor that contributes to the narrowed band gap. The mineralogical characterization including TEM, EDS, and XRF showed that natural goethite contains Al with a doping amount of 1.16–3.23%. The PS analysis showed that the decrease in the band gap of natural goethite is mainly caused by the uplift of the VB. The VB position of natural goethite is −5.06 eV, while that of synthetic goethite is −5.38 eV. The reason behind this phenomenon was revealed by the computational work. The DFT results showed that the VB of goethite is mainly composed of O-2p and Fe-3d orbits. The doping of Al in natural goethite can introduce Al-2p orbits components into the VB, thus elevating the VB position and shorting the forbidden band width. Therefore, electrons can be excited under lower irradiation energy, and natural goethite was proven to have better photocatalytic activity than synthetic goethite in natural exposure environments (Figure 7).

Mineral photocatalysis is one of the most important chemical reactions on the Earth’s surface, and Fe oxide minerals are the most abundant natural semiconducting materials. A qualitative and quantitative understanding of the band structure on natural goethite FeOOH can help us learn how it influences the photochemical process, elements cycling, substance evolution, etc. In the field of semiconducting materials, traditional classical materials such as TiO_2_ and CeO_2_ have been extensively studied due to their good photocatalytic activity. However, the band gap of these materials can be up to 3.0–3.2 eV [32,33], and they can only absorb the ultraviolet spectrum with a wavelength less than 380 nm, which greatly limits their application in the field of photocatalysis. Compared to these synthetic materials, natural goethite has the advantages of longer storage, lower price, and a wider range of light responses. This study’s findings can also provide a good theoretical basis for expanding the application of natural goethite samples in the field of semiconductors.

## 5. Conclusions

Mineralogical characterization showed that natural goethite FeOOH collected from Zhushan Iron Zone commonly occurs in large sizes of 50–300 nm with an Al doping amount of 1.06–3.21%. Therefore, the band gap of the natural goethite sample (2.25 eV) is narrower than that of synthetic goethite (2.55 eV), and the VB position of natural goethite (−5.06 eV) is higher than that of synthetic goethite (−5.38 eV). The theoretical calculation showed this is mainly attributed to the contribution of Al-2p orbits at the topmost part of VB in natural goethite. In light of this, electrons can be excited under lower irradiation energy, and natural goethite demonstrates better photocatalytic performance in MO degradation compared to synthetic goethite. As a ubiquitous phase of Fe oxide minerals, natural goethite shows high efficiency in solar energy utilization, thus having the ability to influence elemental cycling and substance evolution on the Earth’s surface.

## Figures and Tables

**Table 1:** Chemical composition of natural goethite samples.

Sample	Fe_2_O_3_	SiO_2_	Al_2_O_3_	MgO	CaO	K_2_O	Na_2_O	P_2_O_5_	LOI	Total
1	83.17	2.62	1.06	0.11	0.07	0.02	0.12	0.26	11.39	98.82
2	80.79	3.35	1.12	0.13	0.06	0.05	0.00	0.29	12.30	98.09
3	80.57	3.46	1.33	0.11	0.03	0.01	0.02	0.17	12.34	98.04
4	79.91	3.60	1.41	0.18	0.07	0.02	0.18	0.26	12.52	98.15
5	82.60	3.80	2.12	0.02	0.04	0.02	0.06	0.21	8.86	97.73
6	77.71	4.05	3.21	0.05	0.04	0.32	0.00	0.38	11.51	97.27
7	82.28	3.63	1.17	0.01	0.03	0.01	0.18	0.16	10.33	97.80
8	82.93	3.02	1.46	0.04	0.05	0.15	0.05	0.09	10.78	98.57
9	84.79	1.32	2.28	0.02	0.06	0.02	0.03	0.15	10.45	99.12
10	81.52	3.28	1.85	0.14	0.05	0.15	0.00	0.05	10.15	97.19
Average	81.02	3.46	1.79	0.06	0.05	0.02	0.06	0.13	11.13	97.72

**Table 2:** The calculated and experimental cell parameters for goethite.

Values	a (Å)	b (Å)	c (Å)	V (Å^3^)
Calculated	10.02	3.03	4.65	141.18
Experimental	9.91	3.01	4.58	136.62

## Data Availability

Not applicable.

## Supplementary Materials

The following supporting information can be downloaded at: https://www.mdpi.com/article/10.3390/ma15041465/s1, Figure S1: Pure goethite and Al-goethite geometries obtained with GGA + U.
