# Supplementary material for: Determination of Band Structure of Naturally Occurring Goethite with Al Substitution: A Case Study of Zhushan Iron Zone
Source: Materials (Basel). 2022 Feb 16;15(4):1465. doi: 10.3390/ma15041465 (PMC8875212; doi:10.3390/ma15041465)
Supplement: Supplementary file 1 [file materials-15-01465-s001.zip › materials-1590814-supplementary.pdf]

materials

MDPI

Article

Determination of Band Structure of Naturally Occurring Goethite with Al Substitution: A Case Study of Zhushan Iron Zone

Yan Shao [1], Guofeng Hu [1], Zihao Liu [1], Xiaoming Xu [1,2,*], Mengqi Zhang [2], Cong Ding [2] and Yan Li [2]

[1] Wuhan City Environment Protection Engineering Limited Company, Wuhan 430205, China; 37004@ccepc.com (Y.S.); 54001@ccepc.com (G.H.); 10142@ccepc.com (Z.L.)
[2] School of Earth and Space Sciences, Peking University, Beijing 100871, China; mengqizhang@pku.edu.cn (M.Z.); cding_pku@163.com (C.D.); lyanpku@163.com (Y.L.)
* Correspondence: xmxu@pku.edu.cn

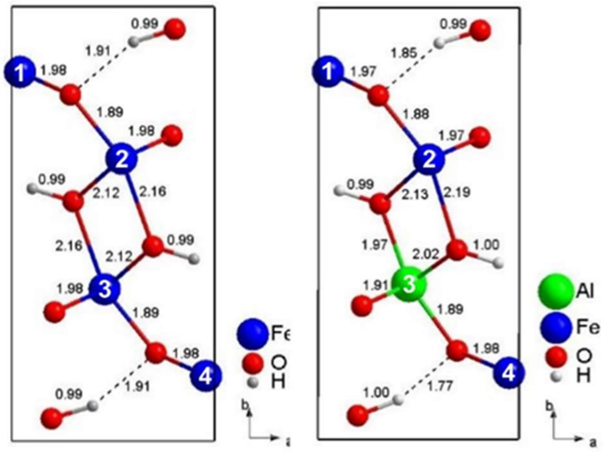

Figure S1. Pure goethite and Al-goethite geometries obtained with GGA+U. Distances between atoms are expressed in Armstrongs (Å).

Citation: Shao, Y.; Hu, G.; Liu, Z.; Xu, X.; Zhang, M.; Ding, C.; Li, Y. Determination of Band Structure of Naturally Occurring Goethite with Al Substitution: A Case Study of Zhushan Iron Zone. *Materials* **2022**, *15*, 1465. https://doi.org/10.3390/ma15041465

Academic Editor: Luminiţa Isac

Received: 25 January 2022
Accepted: 12 February 2022
Published: 16 February 2022

Publisher's Note: MDPI stays neutral with regard to jurisdictional claims in published maps and institutional affiliations.

Copyright: © 2022 by the authors. Submitted for possible open access publication under the terms and conditions of the Creative Commons Attribution (CC BY) license (https://creativecommons.org/licenses/by/4.0/).
